# Prisoners’ Perceptions and Satisfaction with Telepsychiatry Services in Greece and the Effects of Its Use on the Coercion of Mental Healthcare

**DOI:** 10.3390/healthcare12101044

**Published:** 2024-05-18

**Authors:** Evangelia Karachaliou, Phoebe Douzenis, Fotios Chatzinikolaou, Nikos Pantazis, Sophia Martinaki, Panagiota Bali, Konstantinos Tasios, Athanasios Douzenis

**Affiliations:** 1 Psychiatric Hospital of Attica (Dafni), 12462 Chaidari, Greece; 2 University Hospitals of Derby and Burton, Derby DE22 3NE, UK; phoebe28douzenis@gmail.com; 3 Department of Laboratory of Forensic Medicine and Toxicology, School of Medicine, Aristotle University of Thessaloniki, 54124 Thessaloniki, Greece; fotischatzin@auth.gr; 4 Department of Hygiene, Epidemiology and Medical Statistics, School of Medicine, National and Kapodistrian University of Athens, 11527 Athens, Greece; npantaz@med.uoa.gr; 5 Department of Social Work, University of West Attica, 12241 Athens, Greece; smartinaki@uniwa.gr; 6 Second Department of Psychiatry, Medical School, University General Hospital “Attikon”, National and Kapodistrian University of Athens, 12462 Chaidari, Greece; bali_giota@yahoo.com; 7 National Health System, 16672 Athens, Greece; konstantinostasios@gmail.com; 8 Department of Psychiatry, School of Medicine, National & Kapodistrian University of Athens, 15772 Athens, Greece; thandouz@med.uoa.gr

**Keywords:** telepsychiatry, forensic psychiatry, prisons, inmates, coercive measures acceptance, satisfaction

## Abstract

Background: Prisoners are often associated with mental health and substance use disorders. Coercive measures are widely used in prison settings. The objective of this study was to compare inmates’ perceptions and satisfaction with telepsychiatry versus face-to-face consultation and the effects of telepsychiatry on the use of coercive measures. The sample consisted of 100 male inmates from various backgrounds who had experienced both approaches of services (face to face and telepsychiatry). Method: The data were obtained through an interview where the individuals completed a Demographic Data Questionnaire, a Participant Satisfaction Questionnaire to assess satisfaction with face-to-face psychiatric services, and a Participant Satisfaction Questionnaire to assess their satisfaction with services offered via telepsychiatry. Additionally, calculations of time spent waiting for a face-to-face psychiatric evaluation and time spent in handcuffs and in confined spaces were made before and after the introduction of telepsychiatry. Results: Statistically significant improvements (all *p*-values < 0.001) were noted in waiting times, support for relapse prevention, follow up, quality of mental health care, quality of care in the management of psychiatric problems and related medication, behavior of psychiatrists, duration of the assessment, sense of comfort, and confidentiality. Telepsychiatry led to the elimination of time spent in handcuffs and in confined spaces (transport vehicles). Conclusion: According to the results of this study, telepsychiatry is an acceptable method of service delivery in correctional facilities and was associated with a reduction of coercive practices.

## 1. Introduction

Prisoners represent a particularly vulnerable social group with many and complex needs and have a higher incidence of mental health illness and substance use disorders [1,2]. Research studies highlights that, compared to the general population, these individuals suffer from higher rates of mental health problems and substance abuse disorders [2,3,4], higher rates of suicide attempts [5,6] and higher death rates [7], with suicide being the leading cause of death [6]. Moreover, these people are further affected due to comorbid psychiatric and substance use disorders [2,8,9].

It should be noted that these disorders often remain undiagnosed and untreated in correctional settings [10,11]. Factors known to aggravate prisoners’ mental health problems are associated with prison conditions, such as poor living conditions [11], physical [12] and psychological abuse [10], and a shortage of specialized mental health professionals, mainly psychiatrists [13,14,15,16,17,18].

On the other hand, individuals with mental health problems are more vulnerable to a wide range of coercive practices and human rights violations [19]. Coercive measures are widely used in general psychiatry, as well as in forensic and prison psychiatry [20]. They are primarily used to prevent an acute and serious situation from escalating and endangering the life and health of the person subjected to the coercive measures or to people in close proximity to them. In the majority of penitentiary institutions, coercive measures such as handcuffing and keeping are applied without sufficient documentation of their application and usefulness.

The failure of the correctional settings to provide adequate and appropriate psychiatric care results in unmet mental healthcare needs among prisoners [10,11], an infringement of their human rights.

Telepsychiatry is a promising method to address the lack of psychiatric care in correctional facilities. This practice is considered by several researchers as a most relevant, effective, and useful method to help inmates experiencing access problems to specialized psychiatric care [2,13,15,16,17,21,22]. During the COVID-19 pandemic, its use demonstrated increased access to mental health services, effective use by mental health professionals, and a high level of satisfaction among patients and staff [23,24,25,26]. Telepsychiatry services are favored to facilitate and improve access to healthcare services for people living in remote areas or isolated settings [25,26,27].

In Greek prisons, when a person serving his/her sentence presents with a mental health problem, he is not assessed immediately since there is no mental health staff in these facilities. The detainee has to be taken to a local hospital or an appropriate medical facility. Bearing in mind that some prisons are set in a remote area, this requires transporting the prisoner, which is a time-consuming process and requires increased human resources and coordination of different services, as well as increasing the risk of escape or harm to prisoners. This invariably leads to coercive and restrictive measure like handcuffing and guarding them while they wait to be assessed, thus increasing their stigmatization [15,28,29,30].

The use of telepsychiatry reduces the need for transfer of inmates for evaluation to mental health services outside of prisons, as well as reducing their exposure to further coercive measures such as involuntary psychiatric hospitalization and being given involuntary medication [20,31].

In Greece, telepsychiatry as a method to meet the psychiatric care needs of prisoners was introduced in 2018 on a trial basis in four correctional facilities. The aim was to evaluate its appropriateness and potential to tackle the chronic problems observed in the Greek correctional system in relation to inadequate mental health care provided to prisoners. Greece’s correctional system faces major challenges and deficiencies, which, according to the European Committee for the Prevention of Torture—CPT [32], include prison overcrowding, insufficient staffing levels, poor living conditions, inter-prisoner violence, and the absence of an appropriate and integrated management for the provision of health and mental healthcare.

Current protocols for inmates experiencing psychiatric problems (in the acute phase or not) provide for them to be transferred to a local hospital or an appropriate medical facility. This implies transportation over long distances, thus entailing significant costs, cumbersome procedures, increased human resources, and coordination between different administrative bodies, elements which often lead to delays.

The aim of this study is to explore the experiences and level of satisfaction among prisoners in correctional facilities in Greece, with regards to mental health services in the form of telepsychiatry versus conventional face-to-face care as well as the contribution of telepsychiatry in the reduction of restrictive measures. Patient satisfaction is a performance indicator and a way to measure quality of healthcare services [33] and, in particular, of telepsychiatry services [22]. Patient satisfaction with the mental health services provided is important because it results in voluntary participation and commitment to treatment without it being imposed on them.

## 2. Materials and Methods

This study was conducted from September 2020 to February 2021 in Malandrino and Trikala correctional facilities with a capacity of 431 and 600 prisoners, respectively. These facilities launched telepsychiatry services by way of interconnection with the Forensic Psychiatry Unit of the Second Psychiatric Clinic, National and Kapodistrian University of Athens (EKPA), Attikon Hospital.

### 2.1. Sample

The sample of this study consisted of 100 prisoners who were users of mental health services in the two aforementioned correctional facilities. Τhe study sample comprised male prisoners because the implementation of the telepsychiatric services was offered in two male correctional facilities.

On the basis of the selection criteria for the study sample, participants were required (a) to have experience in receiving mental health services both face-to-face in the past and recently, after the covid pandemic, through telepsychiatry, (b) to have attended at least four telepsychiatry sessions, and (c) to have a good knowledge of either Greek or English language. All prisoners who received psychiatric services over the last six months prior to the start of the study were evaluated. A total of 142 individuals were identified. All were approached by the main researcher, and after being informed about the purpose of the study were asked to sign an informed consent form and participate in the study. Of the 142 inmates meeting the above criteria in both correctional facilities, 100 accepted to participate in the study (acceptance rate: 72.42%). A total of 66 were serving their sentences in Malandrino Prison facility and 34 in Trikala.

In order to calculate the time saved under handcuffs and guarding during the “conventional” procedure for psychiatric assessment and treatment, interviews were conducted with the two directors of the correctional facilities that participated in the study in which they were asked to describe in detail the time needed to transfer the prisoners and keep them under guard until they are assessed.

### 2.2. Measurement Instruments

Three questionnaires were distributed and completed via interviews with the participants. Specifically, a (A) Demographic Data Questionnaire, (B) Participant Satisfaction Questionnaire to assess satisfaction with face-to-face psychiatric services, and (C) Participant Satisfaction Questionnaire to assess satisfaction with telepsychiatric services. The construction of the two latter questionnaires was based on an existing questionnaire developed by the Ministry of Health aiming to measure patient satisfaction with services delivered in Greek NHS hospitals. The same questionnaire has also been used in other research studies [34,35] assessing satisfaction among mental health services users in Greece and was standardized in the Greek population [36].

Prior to their use, these questionnaires were modified and adapted to reflect the framework of the correctional facilities.

#### Pilot Study

To establish the acceptability of the questionnaires, a pilot was conducted with the participation of 10 inmates. Participants were selected randomly from the list of inmates that have attended telepsychiatry sessions. Their answers were not included in the final results. This pilot study helped to identify weaknesses and limitations, which then led to the necessary adaptation and determined the final format of the tools used. In particular, the degree of understanding of the questions and the accuracy with which the answers were given were checked in order to identify questions that could lead to biased or vague answers. Following this procedure, it was deemed necessary to provide, in brackets, explanations to certain sub-questions, thereby facilitating the comprehension of these items.

### 2.3. Instrument

From this pilot study, the final Participant Satisfaction Questionnaire was constructed.

(A) The Demographic Data Questionnaire

Consists of nine questions recording data on gender, age, nationality/citizenship, marital status, educational background, and requested reason for assessment.

(B) Participant Satisfaction Questionnaire to assess satisfaction with face-to-face psychiatric services (pre-telepsychiatry implementation)

This questionnaire assessed prisoners’ satisfaction with the delivery of face-to-face psychiatric services. It includes a total of 30 questions, which were measured through a 5-point Likert-type response scale ranging from “very bad” to “very good”. Various factors were used to measure the level of satisfaction: (1) physical environment of the psychiatric assessment room (clean, spacious, quiet, accessibility for persons with disabilities, distractions), (2) service response (waiting time, staffs’ behavior, adequate instructions, appointment time keeping, duration of session, transfer procedure inside the correctional facility), (3) impressions from face-to-face psychiatric assessments (quality, opportunity to express the problem, ease of expression, management of the mental problem offered, reassessment, relapse prevention, e.g., if they themselves thought they had fewer relapse episodes), (4) overall impressions from the delivery of face-to-face psychiatric services delivered (diagnosis and medication offered), (5) assessment of healthcare provided by psychiatrists (how the service users perceived knowledge, abilities, experience, behavior, full briefing about their psychiatric problem), (6) assessment of healthcare provided by health professionals within the correctional facility (responsiveness to the request, behavior, method of implementation of medical instructions), (7) confidentiality and privacy (personal feeling of the participant). The questionnaire included a final question where participants were invited to give an overall evaluation of face-to-face psychiatric services on a rating scale of 0 to 10 and space to provide a free text comment.

(C) Participant Satisfaction Questionnaire to assess satisfaction with telepsychiatric services (post telepsychiatry implementation)

This questionnaire explored the level of satisfaction of participating inmates with the delivery of telepsychiatric services. The questionnaire included the same factors as in the aforementioned questionnaire on face-to-face psychiatric services. Additionally, specific parameters related to telepsychiatry (user-friendly equipment, audio quality, video quality, technical problems) were added, thus increasing the number of questions to 38. Moreover, this questionnaire included two open-ended questions on future attitudes about telepsychiatry in order to assess the prisoner’s preference.

Ιt should be noted that in the questionnaires (pre/post) of our research, there was a clear distinction between the psychiatrist either in the face-to-face contact or via telepsychiatry since these professional were not permanent staff of the Correctional Facilities but of hospitals of the NHS (face-to-face) or university hospital (telepsychiatry).

We considered it appropriate to investigate whether the degree of participants’ satisfaction differed when comparing permanent staff (health professionals) and non-permanent associates (psychiatrists).

(D) Time spent in order for an individual to complete a psychiatric assessment (data from the interviews with the prison directors):

The information received showed the following: Time spent from the Malandrino Correctional Facility to the General Hospital: 90–105 min. Time waiting to be seen: 120 min. Total time spent in handcuffs and being under guard (inc. return journey): on average 360 min (6 h).

Time spent from Trikala Correctional Facility to the General Hospital: 45–60 min. Time waiting to be seen: on average 150 min (less psychiatrists available). Total time spent in handcuffs and being under guard (inc. return journey): on average 300 min (5 h).

### 2.4. Procedure for Completing Questionnaires

A prison officer from each correctional facility coordinated the process of transferring the prisoners from the wards to the interview room provided to the researcher by the directors of the prisons. The researcher first introduced herself to the participants and informed them about the purpose of the research. The participants signed the consent form, which also stated in writing the purpose of the research and the terms of their personal data protection. The researcher administered the *pre* (face-to-face) questionnaire and then the *post* (telepsychiatry) questionnaire for participants to complete. The completion of both questionnaires and the demographic information was carried out through face-to-face interviews with the prisoners without the presence of a prison officer. This helped the prisoners to express their views freely. Also, the presence of the researcher ensured that the questions were understood and ensured that all the questions included in the questionnaires were answered.

### 2.5. Procedure and Ethical Issues 

The study was approved by the General Secretariat for Anti-Crime Policy, Ministry of Citizen Protection, the Scientific Board and the Ethics Board of the General University Hospital “Attikon”, as well as the Board of Directors of the two correctional facilities that took part in the research.

Participants were fully informed about the purpose of the research, their voluntary participation, and their right to withdraw from the research at any time. Informed consent was obtained in writing from all participants involved in the study.

### 2.6. Statistical Methods 

Quantitative variables were summarized using median values and interquartile ranges (IQR), whereas for qualitative ones, absolute (N) and relative (%) frequencies were used. Box-plots were used to visualize the distribution of the variable representing the overall evaluation of psychiatric assessment by the study participants (0–10 scale). For illustrative purposes, questions with possible ordered answers ranging from “very bad” to “very good” were graphically presented using the arithmetic mean of the corresponding values (1: Very bad, 2: Rather bad, 3: Neither good nor bad, 4: Rather good, and 5: Very good) along with the respective 95% confidence intervals of the mean. The above are given separately for the periods before and after the introduction of telepsychiatry.

Changes in the distribution of replies, after the introduction of telepsychiatry, were assessed for statistical significance using appropriate models for repeated measurements. Specifically for questions with ordered (1 to 5) responses, mixed-effects ordinal logistic regression was used, whereas for quantitative variables (i.e., overall evaluation of psychiatric assessment and time gap between requesting to see a psychiatrist and the actual psychiatric session), median regression models with robust standard errors, which took into account the possible correlation between the same person’s responses, were used. Results from mixed-effects ordinal logistic regression are presented as odds ratios for more positive answers after the introduction of telepsychiatry. Odds ratios greater than 1 indicate improvement after the introduction of telepsychiatry and vice versa. Results from quintile regression models are presented as estimated differences in median values after the introduction of telepsychiatry. The same modeling techniques were also used to investigate the effects of other covariates on the overall evaluation of psychiatric assessment and the distribution of the participants’ replies in both periods. Data were managed and analyzed using the statistical software Stata version 15 (Stata Corp., College Station, TX, USA). *p*-values with values less than 0.05 were considered to indicate statistical significance.

## 3. Results

The socio demographic characteristics of participants are summarized in Table 1.

Approximately one-third of the study participants (32%) were sentenced for violent crimes, and more than half of them (55%) had been imprisoned at least once before. 

Participants’ overall perception of the psychiatric assessment process was average for the pre-telepsychiatry period (median 5, IQR 4–6) but increased significantly (*p* < 0.001) by 3 units (95% CI: 1.9–4.1) after the introduction of telepsychiatry, with the corresponding median (IQR) values being 8 (7–9) on the 0–10 scale. The time gap between requesting to see a psychiatrist and the actual psychiatric assessment declined significantly (*p* < 0.001) after the introduction of telepsychiatry from a median (IQR) of 20 (15–22.5) to 9 (5–10) days. 

**Figure 1:**
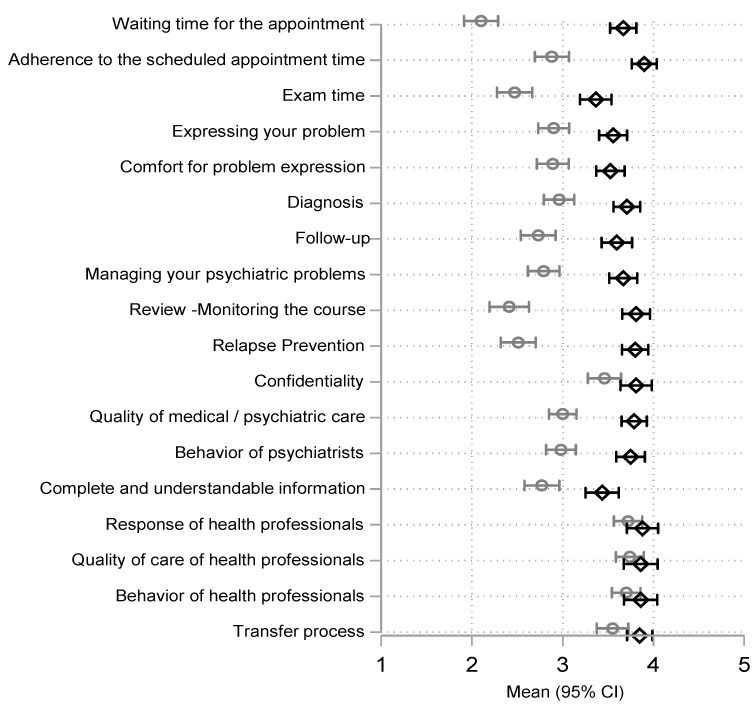
Arithmetic means and respective 95% confidence intervals of replies to questions on satisfaction and acceptance (1 denoting very bad to 5 denoting very good). Gray color indicates replies for the pre-telepsychiatry and black for the telepsychiatry period.

Regarding the remaining questions summarized in Table 2, more positive responses increased significantly after the introduction of telepsychiatry. Responses in these questions are also presented in Table 2 and Figure 1.

From both Table 2 and Figure 1, the greatest improvements were observed for questions related to waiting time for the appointment, relapse prevention, follow up, and scheduled appointment time keeping. Estimated odds ratios for a more positive response after the introduction of telepsychiatry in these questions were all above 10. It is also noteworthy that results indicate substantial improvements in the quality of care, in the management of psychiatric problems and related medication, as well as the behavior of psychiatrists (odds ratios between 6.4 and 8.8). Participants were also significantly more satisfied in terms of duration of the assessment, feeling comfortable when expressing their problems, confidentiality, and transfer process inside the correctional facilities. (odds ratios between 2.4 and 5.3).

Participants’ opinions regarding ease of use of telematics equipment, quality of audio, and quality of video were positive in the vast majority, with the percentages of those replying “rather good” or “very good” ranging from 72% to 80%. Additionally, most of them replied that they would prefer the use of telepsychiatry in the future (69% replied “definitely yes” and 23% “probably yes”). 

Ethnicity of the participants was significantly associated with their overall evaluation of psychiatric assessment. More specifically, participants originating from Asian or North African countries tended to give lower scores in the 0–10 scale by 1 unit on average for both periods (95% CI 0.1 to 1.9 units) compared to Greek participants. 

However, the introduction of telepsychiatry resulted in better overall evaluation of the psychiatric assessment in all ethnic groups, including those from Asian or North African countries, without significant differences in the magnitude of changes between ethnic groups (interaction *p*-value = 0.417). The corresponding results are summarized in Table 3.

Overall evaluation of psychiatric assessment for the pre- or post-telepsychiatry was not significantly associated with age (*p* > 0.999), educational background (*p* = 0.254), marital status (*p* = 0.751), crime type (*p* > 0.999), nor with the existence of previous imprisonments (*p* = 0.325). The aforementioned factors were not associated with the changes in the overall evaluation of psychiatric assessment after the introduction of telepsychiatry either (interaction *p*-values 0.766, 0.289, 0.872, >0.999, 0.949, respectively). 

A similar pattern was observed for participants sentenced for non-violent crimes and the questions related to the expression of their problems, the feeling of comfort when expressing their problems, and the management of their problems. This sub-group had significantly (all *p*-values < 0.01) more positive answers in the period before the introduction of telepsychiatry compared to the subgroup of those sentenced for violent crimes. 

However, the latter group benefited more by the introduction of telepsychiatry, thus differences between the two groups in the telepsychiatry period diminished and became statistically non-significant (all *p*-values > 0.2). 

Finally, according to the official data from the Malandrino and Trikala Prison (interviews with the directors of the correctional facilities), for the transfer of each prisoner for an in-person psychiatric examination from the Malandrino Prison to Lamia General Hospital and Trikala General Hospital and return to the prison, a restraint (handcuffs) was used for 5–6 h. The total time that severe coercive measure was avoided because of the use of telepsychiatry for the 100 participants of the study was 500–600 h. The results are summarized in Table 4.

## 4. Discussion

The aim of this study was the comparative analysis of the factors linked to the satisfaction of individuals serving a prison sentence with regards to face-to-face mental health services and telepsychiatry services and the effects of telepsychiatry on restraint practices. Based on our results, the comparison of the overall satisfaction with the two methods of psychiatric care services showed a significant lead for telepsychiatry. In particular, the median value of the overall evaluation score on a scale of 1–10 for the face-to-face method was “5”, while the corresponding median value for the telepsychiatric method was “8”, indicating a statistically significant improvement (*p* < 0.001) in the overall evaluation of the telepsychiatric service. Similar findings were observed by Guinard et al. [23] in a sample of 3070 patients, in the general population, with different age ranges. The experience using telepsychiatry was either good or excellent for 82.2% of the sample. Farabee et al. [37], in their sample of 71 parolees, found high overall satisfaction with telepsychiatry. Tucker et al. [38], however, found that participating inmates were satisfied with telepsychiatry, but only for specific services. In essence, this means that the individuals that are assessed via telepsychiatry are saving themselves from being under severe coercive measure for 5 to 6 h on the day of the assessment. In this respect, it is not surprising that the intervention studied received very good feedback. A study of 72 patients in a forensic setting found similar scores of satisfaction and outcomes using telepsychiatry as with face-to-face interventions [39]. Furthermore, in a sample of 43 forensic psychiatric patient inmates in a large urban jail who received both telepsychiatry and face-to-face services, Broodey et al. [40] recorded an identical overall satisfaction with psychiatric evaluation for both methods. On the other hand, Slightam et al. [41] found that, when given the option, patients diagnosed with substance use disorder opted for telepsychiatry, a fact fully aligning with our finding due to increased diagnosed rates of the same disorder in our sample of participants. 

In terms of sub-factors, a key element of reference was the waiting time for an appointment that recorded a significant decrease in our sample, namely from 20 to 9 days. Participants’ impressions of appointment waiting time improved with the introduction of telepsychiatry, with the proportion of participants who responded that impressions were “Rather good” or “Very good” increasing from 10% (face-to-face method) to 62% after the introduction of the telepsychiatry method, with the corresponding odds ratios estimated at 26.7 (13.1, 54.2). This shared common ground with various studies [2,15,16,17,22,28,42], all of which point to the fact that telepsychiatry increases specialist accessibility while improving waiting time for psychiatric assessment. In their study, Deslich et al. [28] indicated that inmates have less delay in receiving psychiatric care, while Fox et al. [42] noted a 57% reduction in total waiting time associated with the referral of young inmates. Serhal et al. [43] have shown similar results for patients among the general population.

Increasing prisoners’ access to mental health specialists reduces the use of restrictive methods as the default option when no other support is available [20,31]. Ιn this context, the study by Luxton et Niemi [44] in the United States highlights that court orders for psychiatric forensic evaluations have increased substantially. This has resulted in defendants with mental illness remaining in prison for long periods of time while they await evaluation. Ιn many cases, the symptoms of mentally ill prisoners worsen, and they are subsequently sent to solitary confinement, which contributes to further deterioration of their mental health. This also raises concerns about possible violations of their human rights. The authors concluded that the use of video conferencing (VC) for psychiatric forensic evaluations provided an opportunity to respond to the increased demand of the service by providing additional benefits, leading to a reduction in restrictive practices.

A large proportion of mentally ill people who commit criminal offences tend to be highly resistant to psychiatric treatment [45], failing to comply with psychiatric medication and refusing offered treatment, resulting in the forced imposition of treatment. 

Regarding factors linked to the quality of psychiatric care delivered to participants, the management of their medication, and their psychiatric symptoms as relapse prevention, our sample has demonstrated substantial improvements as a result of telepsychiatry. More specifically, regarding medication management, the percentage of positive impressions of our sample increased from 21% (18% rather good and 3% very good) to 56% (44% rather good and 12% very good) with telepsychiatry. Similarly, regarding the management of their psychiatric symptoms, there was an increase in the percentage of positive impressions from 17% (14% rather good and 3% very good) recorded with the face-to-face method to 60% with telepsychiatry with the corresponding odds ratio estimated at 7.5 (4.1–13.6). A similar pattern was observed in the prevention of relapse, with the percentage of positive impressions increasing from 14% to 67% (52% rather good and 15% very good). These findings are of importance, since the voluntary intake and management of medication leads to better management of psychiatric symptoms and also reduces the risk of relapse and contributes to the reduction in the use of restrictive measures.

In particular, in terms of medication management, our finding is consistent with a 2013 study by Deslich et al. [28], while Farabee et al. [37] fail to confirm this finding. Medication management is a key indicator of the extent to which prison mental health practices are equated with those provided in community settings and that further coercive measures are avoided. Zaylor et al. [46] report that the participants perceived less distress during telepsychiatry treatment, as indicated by decreased self-rated symptom scores over time (*p* < 0.05), which was also in agreement with the psychiatrist impression. Appropriate treatment, frequent monitoring of prisoners, and, in general, mental and emotional stability during imprisonment are essential in avoiding both the relapse of mental illness and the need for the use of coercive methods [31]. Furthermore, Mohr et al. [47] report that the timely provision of treatment and medication to prisoners contributes to relapse prevention and reducing the use of physical restraint and confinement while preventing the occurrence of aggression in the context of different mental disorders. Martin et al. [48] point out that greater satisfaction with treatment increases the likelihood of its continuation, due to the benefit they will have from it in the long term, whereas coercion is associated with treatment dropout and disengagement from mental health services [49].

Ιn line with the results of our study, the percentage of prisoners voluntarily coming for follow up increased with the use of telepsychiatry. Specifically, participants’ impressions improved with the use of telepsychiatry compared to the face-to-face method, with the percentage of positive attitudes increasing from 18% (16% rather good, 2% very good) recorded in the face-to-face method to 67% (50% rather good 17% very good) with the use of telepsychiatry.

When it came to feeling more comfortable when discussing their problems, the therapeutic relationship, and confidentiality, participants showed satisfaction levels confirming previous studies [28,50]. More specifically, regarding the participants’ comfort in discussing their problems, the percentage of positive impressions of the participants increased from 22% (19% rather good and 3% very good) to 48% (36% rather good and 12% very good) with the telepsychiatric method. However, Morgan et al. [51] and Farabee et al. [37], in their studies, found no significant differences between the two methods in terms of working alliance, post-session mood, or overall satisfaction. Confidentiality associated with information dissemination when using telepsychiatry is a major issue in correctional settings. The results of our study record in the factor of confidentiality an increase in the percentage of positive impressions of the participants from 47% (34% rather good and 13% very good) in the face-to-face method to 59% (34% rather good and 25% very good) in the telepsychiatric method. According to existing studies [17,18,21,22,27], low levels of confidentiality in correctional settings are mainly due to the presence of a staff member during the psychological assessment for security reasons, whereas Music [52], in a general population sample linked low confidentiality to the participants’ low educational status. Taking into account the reasons related to security and high rates of low educational attainment, our sample revealed low but satisfactory confidentiality levels. This is also supported by Deslich et al. [28], who pointed out that privacy is not in danger thanks to the use of safe technology, and that this was a source of concern especially among younger people.

In our sample, participants declared they were very satisfied with the user-friendly telematics equipment in combination with separate items such as audio and visual quality. These items occupy an important place in satisfaction expressed by participants and contribute to its overall manifestation [53,54].

A finding of particular importance was linked to the differences among prisoners with a history of violent crimes and those with a history of non-violent crimes. It was found that the former had benefited more from this method and felt more comfortable when expressing themselves, while it also highlighted their need for more sessions and the wider use of this method to include additional services. This subgroup is characterized by a high degree of aggression. This result is considered particularly important because it contributes significantly to the prevention of violent behavior (heterodestructive and self-destructive). The study by Βatastini et al. [2] points out that the use of telepsychiatry contributed to the comparatively lower incidence of disciplinary misconduct among prisoners and the proper management of their psychiatric problems. Similarly, Gómez-Figueroa et al. [55] report that compliance and satisfaction with treatment was associated with a lower risk of violence, which also contributed to a reduction in restraint. Favril et al. [56] highlighted that it has become a global public health priority to determine whether new technologies will work in preventing aggressive behavior in prisoners. Generally, clinicians and patients who used telepsychiatry in addition to the other benefits of the method reported it also contributed to reducing restrictive practices [30].

Also, in agreement with our study, other studies [15,16,17] find that the use of the telepsychiatry method reduces the number of prisoner transfers for psychiatric examination. Towards this direction, an additional important finding of our study is that a significant degree of avoidance of the restrictive measure of transfer and psychiatric examination of handcuffed prisoners results from the reduction in prisoner transfers to a county hospital. It should be noted that the duration of the imposition of this restraint for each in-person psychiatric examination was 5–6 h. The growing awareness of the negative impact of trauma due to the use of restraints is leading to the adoption of a human rights-based framework on the one hand and interventions that minimize them on the other [29].

Our study shows that telepsychiatry led to an improved overall assessment of the psychiatric service in all ethnic groups, including participants of Asian, North African, and Greek descent. Foreign prisoners are subject to deprivation of liberty and criminal proceedings in a foreign state of which they are neither nationals nor residents. Imprisonment is another potentially traumatic event characterized by further difficulties due to language difficulties and cultural differences [57]. According to studies, individuals with mental illness who come from racial-ethnic minorities often experience “double stigma,” or discrimination associated with living with a mental illness combined with racial-ethnic discrimination, which results in people either avoiding seeking treatment or refusal of treatment, which can lead to it being imposed on them in prison contexts [58,59]. Therefore, their satisfaction with telepsychiatry demonstrated that they could more easily and voluntarily seek out psychiatric treatment. 

Finally, regarding the future preferences of the participants, it should be noted that the vast majority of participants in our sample 92% (21% rather yes and 71% definitely yes) indicated they would recommend telepsychiatry to fellow inmate and expressed their desire to continue to receive telepsychiatry services. This, in our opinion, is connected with the avoidance of being in handcuffs and guarded for 5–6 h on the day of the assessment. 

## 5. Strengths and Limitations

This study is the first to be carried out in Greece since the introduction of telepsychiatry in correctional facilities. Despite its strengths, such as racial and cultural diversity, it also includes a series of gender-based limitations, as only male prisoners are housed in these facilities. These do not allow us to draw comparative data in terms of gender. Other limitation includes the lack of authorization to study the legal files of the inmates kept in each correctional facility.

Also, the data show that people of color and people from minority ethnic backgrounds are overrepresented in criminal justice services [60], and according to our findings, they had lower satisfaction rates with both methods, which suggests that they probably value the care situation differently. Further research is needed to examine cultural and ethnic differences in experience and satisfaction.

Finally, there is a need for long-term follow up and further research regarding the clinical results of the telepsychiatry intervention and the investigation of the degree of satisfaction over time.

## 6. Conclusions

The findings in this study have produced evidence from the introduction of telepsychiatry in correctional facilities about acceptability and satisfaction with telepsychiatry. Though there were sample nuances in the findings, the prisoners’ overall satisfaction highlights the need to expand telepsychiatry in an effort to meet the needs of this vulnerable population group of prisoners.

According to the results of our study, the improvement observed in the satisfaction factors under consideration like the comfort in expressing the problem, follow up, medication management, treatment, psychiatric symptom management, and relapse prevention demonstrated the contribution of telepsychiatry in reducing restraints, while in the long term it could contribute to reducing offending behavior and reducing the incarceration rate. 

In addition, the use of telepsychiatry in prisoners can serve as an equalizing force in psychiatric care, mitigating initial inequalities linked to ethnicity on the one hand and restrictive practices and violation of their human rights on the other.

The findings may also be used as a strong encouragement for policy makers to bridge the gap between psychiatric care supply and demand for prisoners and to reduce the use of restrictive practices in correctional facilities. Further research and evidence are needed with regards to the long-term impact of telepsychiatry on the use of coercive measures in prisons, but so far, the evidence appears to suggest that it has a positive impact on reducing the need and application of these coercive measures. 

This is in line with the international standards and human rights regarding the dignity and equality of services offered to the prison population, ensuring equal value and status for prisoners with mental illness.

## Figures and Tables

**Table 1:** Demographic characteristics and sentences of study participants (N = 100).

Variable	Overall
Sex	
– Male	100 (100.00%)
Age–Median (IQR)	38.0 (30.0, 44.0)
Origin	
– Greece	59 (59.00%)
– N. Africa/Asia	28 (28.00%)
– Eastern Europe	13 (13.00%)
Education level	
– Graduate of primary school	38 (38.00%)
– High school (gymnasium) graduate	17 (17.00%)
– High school (lyceum) graduate	4 (4.00%)
– Graduate of higher education	1 (1.00%)
– Illiterate (individuals that have not completed the 6 grades of primary school)	40 (40.00%)
Type of offense	
– Violent	32 (32.00%)
– Non-violent	60 (60.00%)
– Non-response	8 (8.00%)
Years of sentence–Median (IQR)	15.0 (10.0, 20.0)
First time serving a sentence	
– Yes	24 (24.00%)
– No	55 (55.00%)
– I do not answer	18 (18.00%)
– Defendant	3 (3.00%)
If not, how many times have you served a sentence–Median (IQR)	2.0 (2.0, 4.0)

**Table 2:** Degree of satisfaction and acceptance. Figures are median (IQR) for quantitative variables and N (%) of positive responses (rather good or very good) for qualitative variables. Estimated effect sizes are differences in median values (quantitative variables—median regression) or odds ratios for more positive responses (qualitative variables—ordinal logistic regression using the full 1–5 scale of responses) after the introduction of telepsychiatry.

Variable	Pre	Post	Estimated Effect Size (95% CI)	*p*-Value
Time gap (days)	20.0 (15.0, 22.5)	9.0 (5.0, 10.0)	−11.0 (−13.6, −8.4)	<0.001
Waiting time for the appointment	10 (10.00%)	62 (62.00%)	26.7 (13.1, 54.2)	<0.001
Adherence to the scheduled appointment time	28 (28.00%)	77 (77.00%)	10.4 (5.6, 19.4)	<0.001
Exam time (duration of mental health assessment)	16 (16.00%)	42 (42.00%)	5.3 (3.1, 9.3)	<0.001
Expressing your problem	22 (22.00%)	48 (48.00%)	4.8 (2.6, 8.9)	<0.001
Comfort for problem expression	22 (22.00%)	46 (46.00%)	4.5 (2.5, 8.2)	<0.001
Diagnosis	20 (20.00%)	61 (61.00%)	6.8 (3.5, 13.3)	<0.001
Medication recommendation/management	21 (21.00%)	56 (56.00%)	6.7 (3.6, 12.4)	<0.001
Managing your psychiatric problems	17 (17.00%)	60 (60.00%)	7.5 (4.1, 13.6)	<0.001
Follow up	18 (18.00%)	67 (67.00%)	13.3 (7.1, 24.8)	<0.001
Relapse prevention	14 (14.00%)	67 (67.00%)	15.9 (8.2, 30.7)	<0.001
Confidentiality	47 (47.00%)	59 (59.00%)	3.0 (1.6, 5.5)	0.001
Quality of medical/psychiatric care	21 (21.00%)	68 (68.00%)	8.8 (4.4, 17.6)	<0.001
Behavior of psychiatrists	24 (24.00%)	63 (63.00%)	6.4 (3.4, 12.4)	<0.001
Complete and understandable information	21 (21.00%)	47 (47.00%)	3.9 (2.2, 7.0)	<0.001
Response of health professionals	68 (68.00%)	70 (70.00%)	1.8 (1.0, 3.4)	0.051
Quality of care of health professionals	68 (68.00%)	68 (68.00%)	1.6 (0.9, 2.8)	0.109
Behavior of health professionals	65 (65.00%)	67 (67.00%)	1.7 (1.0, 3.1)	0.058
Transfer process	59 (59.00%)	72 (72.00%)	2.4 (1.3, 4.3)	0.004
Overall assessment of psychiatric examination	5.0 (4.0, 6.0)	8.0 (7.0, 9.0)	3.0 (1.9, 4.1)	<0.001

**Table 3:** Overall assessment of psychiatric examination by ethnicity.

Variable	Pre	Post	Overall
	n = 100 (50.00%)	n = 100 (50.00%)	N = 200 (100%)
Greece–Median (IQR)	6.0 (5.0, 6.0)	8.0 (7.0, 9.0)	7.0 (5.0, 8.0)
N. Africa/Asia–Median (IQR)	4.0 (4.0, 5.0)	7.0 (6.0, 9.0)	6.0 (4.0, 7.0)
Eastern Europe–Median (IQR)	6.0 (5.0, 6.0)	8.0 (7.0, 9.0)	6.0 (5.0, 8.0)
Overall–Median (IQR)	5.0 (4.0, 6.0)	8.0 (7.0, 9.0)	6.0 (5.0, 8.0)
Estimated (post–pre) difference (95% CI) for all groups	3.0 (2.2, 3.8)	*p*-value < 0.001	
Estimated (N. Africa/Asia–Greece) difference (95% CI) for both pre and post	−1.0 (−1.9, −0.1)	*p*-value = 0.024	
Estimated (Eastern Europe–Greece) difference (95% CI) for both pre and post	0.0 (−1.2, 1.2)	*p*-value > 0.999	
Interaction ethnicity X pre–post		*p*-value = 0.417	

**Table 4:** Duration of the use of restrictive measures (use of handcuffs) for the transfer of a prisoner for psychiatric examination by the face-to-face method.

**Measurement Unit (One Face-to-Face Psychiatric Examination of One Inmate**	**Transfer from Malandrino to General Hospital (Psychiatric Examination**	**Time Waiting**	**Time of Psychiatric Examination**	**Transfer from General Hospital to Maladrino Prison**	**Total Time (in Minutes) of Use of Handcuffs**
1 inmate	90–105 min	120	30	90–105	330–360 min
**Measurement unit** **(One face-to-face psychiatric examination of one inmate**	**Transfer from Trikala Prison to General Hospital (Psychiatric Examination**	**Time waiting**	**Time of Psychiatric Examination**	**Transfer from General Hospital to Trikala Prison**	**Total Time (in Minutes) of Use of Handcuffs**
1 inmate	45–60	150	30	45–60	270–300

## Data Availability

Data are not shared due to ethical issues.
